# Study on the Regulation of Exogenous Hormones on the Absorption of Elements and the Accumulation of Secondary Metabolites in the Medicinal Plant *Artemisia argyi* Leaves

**DOI:** 10.3390/metabo12100984

**Published:** 2022-10-17

**Authors:** Linlin Yang, Yueci Yan, Boyu Zhao, Huaming Xu, Xiuhong Su, Chengming Dong

**Affiliations:** 1 School of Pharmacy, Henan University of Chinese Medicine, Zhengzhou 450046, China; 2 Henan Provincial Ecological Planting Engineering Technology Research Center of Daodi Herbs, Henan University of Chinese Medicine, Zhengzhou 450046, China; 3 Co-Construction Collaborative Innovation Centre for Chinese Medicine and Respiratory Diseases by Henan & Education Ministry of China, Henan University of Chinese Medicine, Zhengzhou 450046, China

**Keywords:** *Artemisia argyi*, exogenous hormones, elements, secondary metabolites

## Abstract

As an important medicinal plant, we still do not know the effect of exogenous hormones on absorption of elements and accumulation of secondary metabolites in *Artemisia argyi* leaves. In this work, we analyzed the difference in 21 elements absorbed by *A. argyi* leaves under three exogenous hormone (MeJA, SA and ABA) treatments, and also clarified the correlation between 21 elements and eight bioactive components. Different hormone treatments changed the absorption and enrichment of elements, and the composition also changed significantly. The contents of eight bioactive components changed significantly under different hormone treatments. When *A. argyi* was stimulated by exogenous hormones, the content of secondary metabolites was adjusted in the leaves through changes in the absorption and enrichment of elements. The widely untargeted metabolomic analysis further confirmed that ABA changes the metabolic direction of secondary metabolites in *A. argyi* leaves and stimulates the biosynthesis of multiple secondary metabolites including phenylpropanoids, flavonoids, terpenoids, alkaloids and others. These results provide a new perspective for the changes in element absorption and the mechanism of secondary metabolic components in *A. argyi* leaves under exogenous hormone treatments, and also deepen people’s understanding of the interaction mechanism between medicinal plants and hormones.

## 1. Introduction

*Artemisia argyi* (Compositae) is a perennial herb or a semi half-shrub-like plant with a high aroma volatiles [1], which is the origin plant of *Artemisia argyi* Folium in Chinese traditional medicine [2]. *A. argyi* has a wide distribution range, in addition to extremely arid and alpine regions, of which there are several throughout China [3]. *A. argyi* mainly grows on wasteland, roadsides, riversides and hillsides in low- to medium-altitude areas. It is also found in forest, and grassland areas [4]. In some areas, *A. argyi* is the dominant species in plant communities. The whole herb of *A. argyi* can be used as medicine to warm meridians, remove dampness, dissipate cold, stop bleeding, inhibit inflammation, relieve asthma, prevent miscarriage and resist allergy [5,6,7]. *Artemisia argyi* Folium is recorded in medical books of past dynasties as “essential medicine for hemostasis” [8], and is one of the common gynecological drugs, especially for the treatment of gynecological diseases of deficiency and cold [9], and also for the treatment of chronic bronchitis and asthma in the elderly [10]. In addition, the whole herb of *A. argyi* is used as an insecticidal pesticide or fumigation for rooms [11]. Due to its rich convalescent application value, with the development of the health industry, people pay more attention to recuperation and health care, and *A. argyi* leaves are more popular in the daily life of the public [12]. Therefore, it is particularly important to carry out basic research on the medicinal botany of *A. argyi*.

Elements are the material basis for plant growth and development and the formation of nutritional quality [13]. Studies have shown that the content and proportion of elements in plants can affect the synthesis and accumulation of bioactive components in *Paeonia lactiflora* [14], *Astragalus membranaceus* [15], *Salvia miltiorrhiza* [16] and other medicinal plants. Benzoic acid concentration in *P. lactiflora* showed positive linear dependence upon the contents of P and Mn. In *A. membranaceus*, there was a strong correlation between elements and active components such as Ba-astragaloside IV, Co-astragaloside IV, Zn-calycosin and Li-formononetin. The leaves are the main medicinal parts of *A. argyi* and contain abundant medicinal bioactive components [17]. The content of elements in leaves of *A. argyi* can represent the utilization of nutrients in plants, and it is the expression of elements in soil absorbed by *A. argyi*. The altered chemical composition in *A. argyi* leaves may be the result of absorption of different elements in the soil, thus clarifying the relationship between elements, and chemical composition is of applied value to improve mugwort cultivation practices. At present, the research on *A. argyi* is mainly focused on its medicinal bioactive components and pharmacological activities. There are few reports on the research of elements other than the main secondary metabolites in *A. argyi* leaves, and the interaction between elements and the main secondary metabolites in *A. argyi* leaves is also unclear. Therefore, it is particularly important to study the correlation between the content and composition of elements and the secondary metabolites in *A. argyi* leaves.

Plant hormones play an important role in each stage of plant growth and development, regulating plant development and secondary metabolism [18]. Plant hormones can be divided into six categories: auxin, cytokinin, abscisic acid (ABA), gibberelin (GA), ethylene and brassinolide (BR) [19]. In recent years, many new plant hormones such as jasmonic acid (JA) and salicylic acid (SA) have been found [20]. Plant hormones can not only regulate plant development, metabolism, aging and other physiological processes by affecting nucleic acid, protein and enzymes in plants, but also regulate the synthesis of flavonoids, terpenoids, alkaloids and other secondary metabolites in plants [21]. For example, exogenous ABA at an appropriate concentration can promote the accumulation of the main bioactive components (glycyrrhizin, liquiritin, isoliquiritin, liquiritigenin and isoliquiritigenin) in *Glycyrrhiza uralensis* [22]. Exogenous ABA can also regulate saikosaponin synthesis of *Bupleurum chinense* under drought stress [23]. Other researchers found that after induction by JA, callus cultures of *Hibicus sabdariffa* accumulated more bioactive compounds and showed stronger antioxidant activity [24]. Salicylic acid acted as an elicitor to stimulate the accumulation of phenols and flavonoids in callus culture of *Ruta graveolens* [25]. So, it is unclear whether exogenous hormones can affect the synthesis of plant secondary metabolites by changing the absorption and enrichment of elements. To date, no research has been carried out on *A. argyi* to answer this scientific question.

In order to reveal whether exogenous hormones affect the absorption of elements and change the synthesis and accumulation of secondary metabolites, we carried out this study on the regulation of exogenous hormones on the absorption of elements and the accumulation of secondary metabolites in leaves of the medicinal plant *A. argyi*. In this work, three exogenous hormones (MeJA, SA and ABA) were used to treat *A. argyi* leaves. We analyzed the difference in 21 elements absorbed by *A. argyi* leaves under three exogenous hormone treatments, and also clarified the correlation between 21 elements and eight bioactive components. The mechanism of the effect of ABA on the formation of secondary metabolic components in *A. argyi* leaves was further investigated by widely untargeted metabolomic analysis. This study provides a new perspective for the changes in element absorption and the mechanism of secondary metabolic components in *A. argyi* leaves under exogenous hormone treatments, and also deepens people’s understanding of the interaction mechanism between medicinal plants and hormones.

## 2. Materials and Methods

### 2.1. Experimental Materials and Treatment Methods

*Artemisia argyi* plants were collected in the medicinal botanical garden of Henan University of Chinese Medicine, Zhengzhou, Henan Province, China. The vigorous, disease-free *A. argyi* plants were transplanted into plastic pots (the specification was 25 cm in diameter and 30 cm in height, holes in the bottom of pot and two plants per pot). Standardized field management to was carried out ensure proper growth of *A. argyi* plants and experimental treatments were started when plants reached 120 cm in height (60 days). A total of 100 pots of *A. argyi* plants were used in the experiment and were divided into 4 groups according to different treatments (*n* = 25). The methyl jasmonate (MeJA) treatment group: at 18:00 every day, plant leaves were sprayed with 1 L MeJA at a concentration of 5 mg·L^−1^ for three consecutive days. The salicylic acid (SA) treatment group: at 18:00 every day, plant leaves were sprayed with 1 L SA at a concentration of 5 mg·L^−1^ for three consecutive days. The abscisic acid (ABA) treatment group: at 18:00 every day, plant leaves were sprayed with 1 L ABA at a concentration of 5 mg·L^−1^ for three consecutive days. The control group (CK): at 18:00 every day, plant leaves were sprayed with 1 L of distilled water for three consecutive days. The experiment cycle was 30 days. At the end of the experiment, fresh *A. argyi* leaves were collected, some of which were dried in the shade for ICP-MS and HPLC analysis, and the rest were stored in an ultra-low-temperature refrigerator at −80 °C for widely untargeted metabolome analysis.

### 2.2. Determination of 21 Element Contents in A. argyi Leaves by ICP-MS

Preparation of the sample solution: the blade of the sample to be tested was pulverized by a high-speed pulverizer, passed through a 10 mesh sieve and the sample was accurately weighed and 0.2 g was digested according to the standard operation procedure of the MARS 2 microwave digestion instrument (CEM Corporation, Matthews, NC, USA). The working conditions of the microwave digestion instrument (power 1600 kW) were as follows: (1) heating time 8 min, temperature 100 °C, holding time 5 min; (2) heating time 5 min, temperature 150 °C, holding time 5 min; (3) heating time 2 min, temperature 170 °C, holding time 5 min; (4) heating time 2 min, temperature 190 °C, holding time 12 min. After digestion, the digestion solution was transferred to a 50 mL volumetric flask and the volume was increased with ultra-pure water to obtain the sample solution for subsequent analysis.

Preparation of mixed standard solution: a standard solution of mixed reference containing 21 elements (^11^B, ^24^Mg, ^27^Al, ^31^P, ^39^K, ^44^Ca, ^48^Ti, ^51^V, ^52^Cr, ^55^Mn, ^57^Fe, ^59^Co, ^60^Ni, ^63^Cu, ^75^As, ^77^Se, ^88^Sr, ^95^Mo, ^111^Cd, ^137^Ba and ^208^Pb) was precisely absorbed and diluted to a concentration of 100 ppb as a mixed standard solution.

ICP-MS working conditions: power 1548.60 w, cooling gas flow 13.92 L·min^−1^, auxiliary gas flow 0.80 L·min^−1^, atomization gas flow 0.99 L·min^−1^, collision reaction gas was helium, collision reaction gas flow 0.50 L·min^−1^, concentric atomizer, sampling cone was nickel cone, scanning times 3, residence time 30 ms, sampling depth 5 mm.

Linear relationship: standard solutions with concentrations of 0, 0.1, 0.5, 1, 5, 10, 25, 50 and 100 ppb were prepared by dilution with 5% HNO_3_ solution. Taking 10 ppb ^6^Li, ^73^Ge, ^115^In and ^209^Bi as internal standards, the element content was determined according to the working conditions of ICP-MS. The standard curve was drawn with peak area as abscissa and concentration as ordinate. The linear relationship is shown in Table S1.

Content determination: according to the above method, 21 elements in the sample solution were determined and their contents in the *A. argyi* leaves were calculated according to the linear relationship. All samples were biologically repeated three times.

### 2.3. Determination of Eight Bioactive Components in A. argyi Leaves by HPLC

Preparation of the sample solution: the samples to be measured were precisely weighed at 0.5 g, placed in a 50 mL conical flask with stopper and 15 mL 75% methanol was added. The conical flask was treated with an ultrasonic extractor (power 250 W, frequency 100 kHz) for 30 min. When the conical flask was cooled to room temperature, the extract was filtered through quantitative filter paper to obtain the sample solution.

Preparation of mixed standard solution: 1 mg of neochlorogenic acid, chlorogenic acid, isochlorogenic acid A, isochlorogenic acid B, isochlorogenic acid C, 7-hydroxycoumarin, jaceosidin and eupatilin standards was weighed with an analytical balance, dissolved with methanol and added to a 5 mL volumetric flask. A mixed standard solution containing the above components with a mass concentration of 0.02 mg·mL^−1^ was prepared.

Chromatographic conditions: a Waters 2695 liquid chromatograph system (Waters Corporation, Milford, MA, USA) equipped with a vacuum degasser, a quaternary gradient pump, an auto sampler and a diode array detector attached to an Agilent ZORBAX SB-C18 column (4.6 mm × 150 mm, 5 μm). The binary gradient elution system consisted of acetonitrile (A) and 0.1% formic acid aqueous solution (B), and separation was achieved using the following gradient program: 0–5 min, 10% A; 5–10 min, 20% A; 10–18 min, 31% A; 18–28 min, 45% A; 28–38 min, 60% A. The column temperature was 25 °C. The detection wavelength was 340 nm. The flow rate was 0.8 mL·min^−1^. The injection volume was 10 μL.

**Figure 1:**
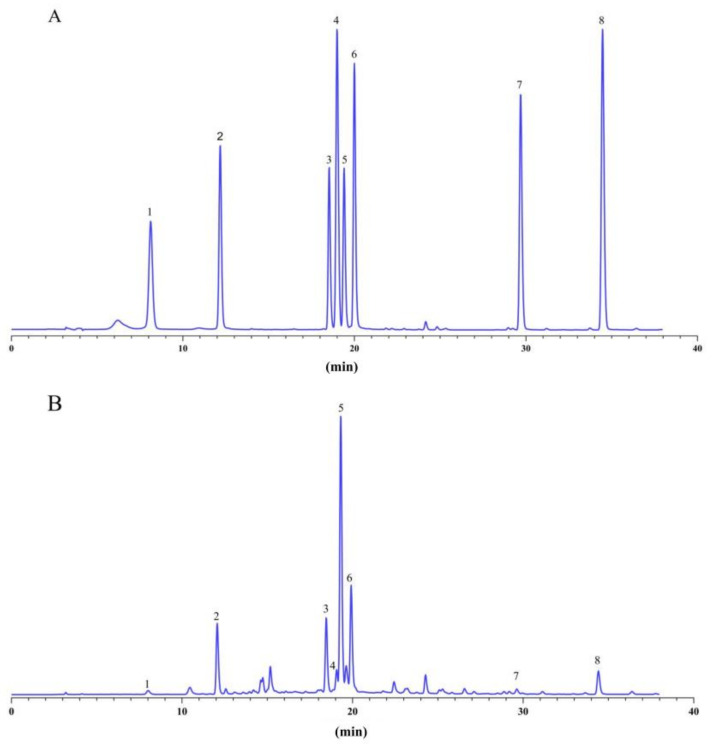
HPLC chromatogram of mixed standard solution (**A**) and sample solution (**B**); 1: neochlorogenic acid; 2: chlorogenic acid; 3: isochlorogenic acid B; 4: 7-hydroxycoumarin; 5: isochlorogenic acid A; 6: isochlorogenic acid C; 7: jaceosidin; 8: eupatilin.

Content analysis: seven concentrations of 8 analytes were injected, and then calibration curves were constructed by plotting the peak area versus the concentration of each analyte. The chromatograms of mixed standard solution and sample solution are shown in Figure 1. The linear relationship is shown in Table S2. The sample solution passed through a 0.22 μM organic microporous membrane filter, and the filtrate was analyzed under the above chromatographic conditions. Combined with the HPLC results and linear relationship, the contents of 8 bioactive components were calculated. All samples were biologically repeated three times.

### 2.4. Widely Untargeted Metabolomic Analysis of A. argyi Leaves

In order to further explain the effect of ABA treatment on the *A. argyi* leaves’ metabolite changes, we used a widely untargeted metabolomic analysis to analyze the changes in metabolites in ABA treatment (*n* = 6) and CK (*n* = 6). This analysis was carried out on an ultra-performance liquid chromatography (UPLC)–MS/MS system: a UPLC (UltiMate 3000 HPLC, Thermo Fisher Scientific, San Jose, CA, USA) connected to a high-resolution tandem mass spectrometer (Q-Exactive, Thermo Fisher Scientific, Saint Louis, MO, USA). This widely untargeted metabolomic analysis was completed by LC-Bio Technologies Co., Ltd. (HangZhou, China). Basic data were collected and identified using the methods previously reported [26]. The open source software metaX was used to analyze the metabolomic dataset, and univariate and multivariable analyses were performed to obtain the differentially accumulated metabolites (DAMs) between ABA and CK.

### 2.5. Statistical Analysis

The final experimental data with single-factor variance analysis and correlation analysis were analyzed by SPSS software (version 19.0, SPSS Inc., Chicago, IL, USA). GraphPad Prism Software (version 9.0, GraphPad Software Inc., San Diego, CA, USA) was used to draw the graphics of element content and content of 8 bioactive components. Bioinformatic analysis-related widely untargeted metabolomic analysis was performed using the OmicStudio tools (https://www.omicstudio.cn/tool) (accessed on 15 July 2022).

## 3. Results

### 3.1. The Content of 21 Element Contents in A. argyi Leaves

**Figure 2:**
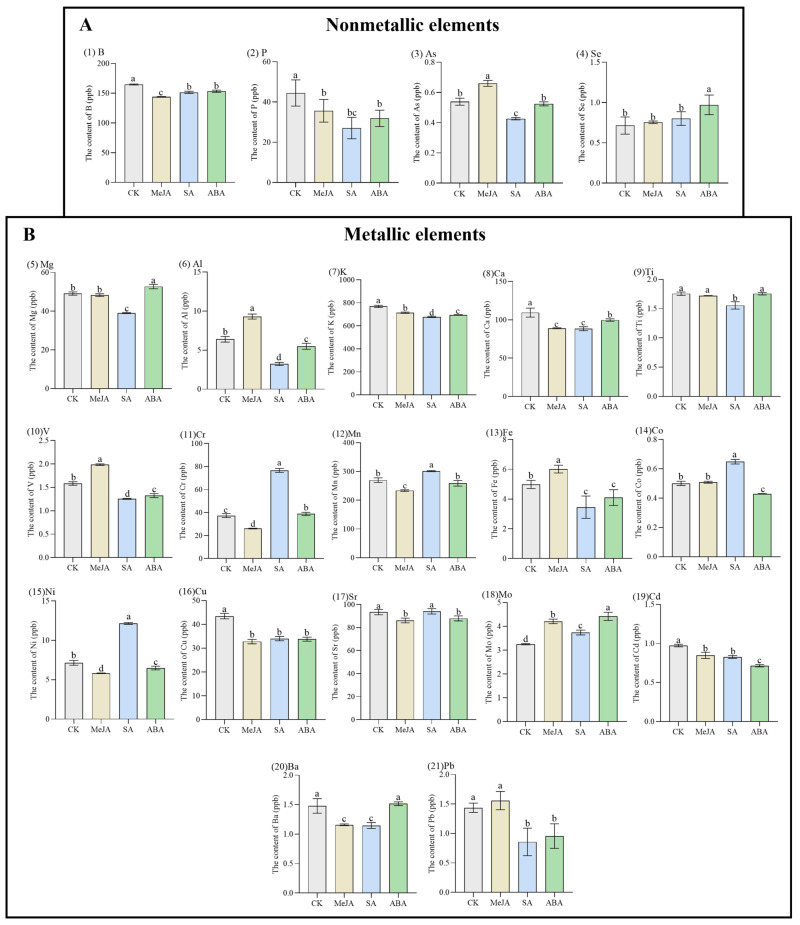
Contents of nonmetallic elements (**A**) and metal elements (**B**) in *A. argyi* leaves. The data are expressed as the means ± standard deviations (SDs, *n* = 3). The different lowercase letters indicate significant differences between treatments (*p* < 0.05) by Duncan’s single-factor variance analysis.

The contents of 21 elements (including 4 non-metallic elements and 17 metal elements) in *A. argyi* leaves changed significantly under different hormone treatments (MeJA, SA and ABA). The result is shown in Figure 2. For the four nonmetallic elements (B, P, As and Se), the contents of B and P decreased significantly under different hormone treatments (*p* < 0.05), compared with CK, while the content of As increased significantly after MeJA treatment (*p* < 0.05), and the content of Se increased significantly after ABA treatment (*p* < 0.05).

As shown in Figure 2B, the contents of 17 metal elements changed significantly under different hormone treatments. The content of Mg after ABA treatment was 52.76 ppb, significantly higher than CK (*p* < 0.05). The content of Al after MeJA treatment was 9.30 ppb, significantly higher than CK (*p* < 0.05). The content of Al after MeJA treatment was 9.30 ppb, significantly higher than CK (*p* < 0.05). The contents of K, Ca, Cu and Cd decreased significantly under different hormone treatments (*p* < 0.05). The content of Ti decreased significantly only after SA treatment, compared with CK (*p* < 0.05). The content of V after MeJA treatment was 1.99 ppb, significantly higher than CK (*p* < 0.05). The content of Cr after SA treatment was 76.62 ppb, significantly higher than CK (*p* < 0.05). The content of Mn after SA treatment was 301.20 ppb, significantly higher than CK (*p* < 0.05). The content of Fe after MeJA treatment was 6.02 ppb, significantly higher than CK (*p* < 0.05). The content of Co after SA treatment was 0.65 ppb, significantly higher than CK (*p* < 0.05). The content of Ni after SA treatment was 12.15 ppb, significantly higher than CK (*p* < 0.05). The content of Sr decreased significantly after MeJA and ABA treatment, compared with CK (*p* < 0.05). The content of Mo after ABA treatment was 4.42 ppb, significantly higher than CK (*p* < 0.05). The content of Ba decreased significantly after MeJA and SA treatment, compared with CK (*p* < 0.05). The content of Pb decreased significantly after SA and ABA treatment, compared with CK (*p* < 0.05). It can be seen that different hormone treatments changed the absorption of various elements in *A. argyi* leaves, which may further affect the synthesis of secondary metabolism in plant tissues.

**Figure 3:**
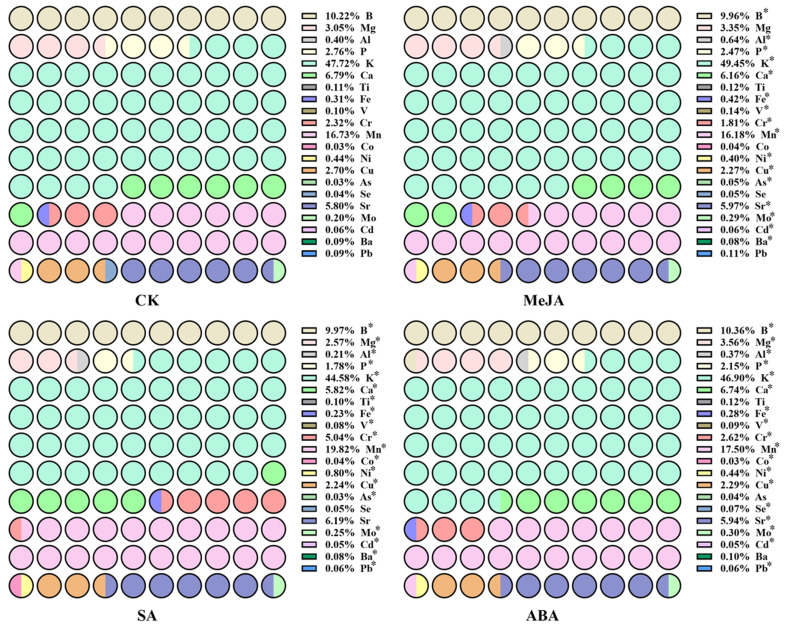
The proportion of 21 elements in *A. argyi* leaves under the CK, MeJA, SA and ABA treatments. * indicates that the treatment group and control group significantly differed at the 0.05 level.

We further analyzed the proportion of 21 elements in *A. argyi* leaves, and the results are shown in Figure 3. K, Mn, B, Ca and Sr were the top five elements in the leaves of *A. argyi*. In CK, the proportions of K, Mn, B, Ca and Sr were 47.72%, 16.73%, 10.22%, 6.79% and 5.80%, respectively. In the MeJA treatment group, the proportions of K, Mn, B, Ca and Sr were 49.45%, 16.18%, 9.96%, 6.16% and 5.97%, respectively. In the SA treatment group, the proportions of K, Mn, B, Ca and Sr were 44.58%, 19.82%, 9.97%, 5.82% and 6.19%, respectively. In the ABA treatment group, the proportions of K, Mn, B, Ca and Sr were 46.90%, 17.50%, 10.36%, 6.74% and 5.94%, respectively. The analysis shows that different hormone treatments affect the absorption of various elements by *A. argyi* leaves, thus changing the proportion of various elements in tissues.

### 3.2. The Content of Bioactive Components in A. argyi Leaves

**Figure 4:**
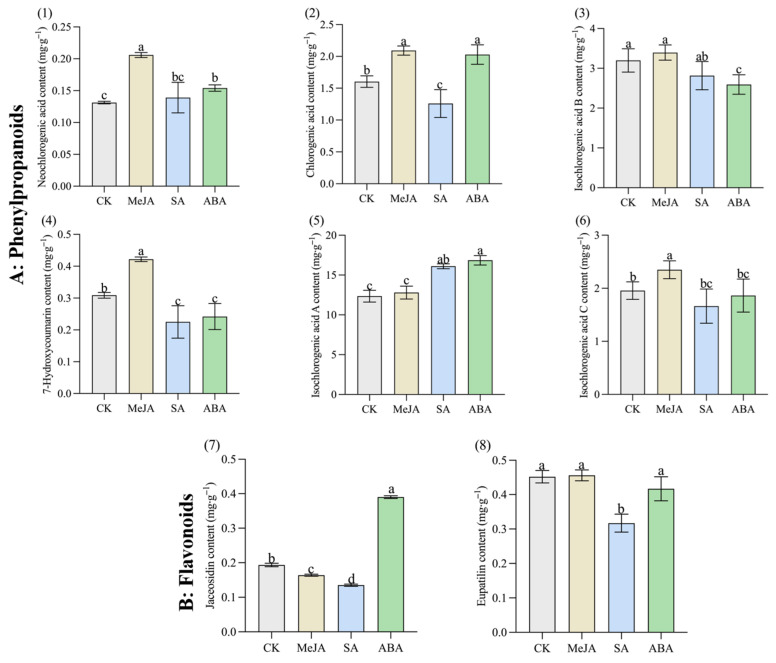
Changes in the content of eight bioactive compounds (six phenylpropanoids and two flavonoids) of *A. argyi* leaves under the CK, MeJA, SA and ABA treatments. (**1**) Neochlorogenic acid; (**2**) chlorogenic acid; (**3**) isochlorogenic acid B; (**4**) 7-hydroxycoumarin; (**5**) isochlorogenic acid A; (**6**) isochlorogenic acid C; (**7**) jaceosidin; (**8**) eupatilin. The data are expressed as the means ± standard deviations (SDs, *n* = 3). The different lowercase letters indicate significant differences between treatments (*p* < 0.05) by Duncan’s single-factor variance analysis.

Different hormone treatments also affected the content of bioactive components in *A. argyi* leaves. The changes in eight bioactive components (six phenylpropanoids and two flavonoids) are shown in Figure 4. After MeJA treatment, the contents of neochlorogenic acid, chlorogenic acid, 7-hydroxycoumarin and isochlorogenic acid C reached 0.206 mg·g^−1^, 2.092 mg·g^−1^, 0.422 mg·g^−1^ and 2.349 mg·g^−1^, respectively, which were significantly increased by 57.01%, 30.42%, 36.66% and 20.04% compared with CK (*p* < 0.05), respectively. After SA treatment, only the content of isochlorogenic acid A was significantly increased by 30.41%, compared with CK (*p* < 0.05), the content was 16.105 mg·g^−1^. The content of other bioactive components decreased in varying degrees after SA treatment. After ABA treatment, the contents of neochlorogenic acid, chlorogenic acid, isochlorogenic acid A and jaceosidin reached 0.154 mg·g^−1^, 2.029 mg·g^−1^, 16.852 mg·g^−1^ and 0.390 mg·g^−1^, respectively, which were significantly increased by 17.38%, 26.50%, 36.46% and 101.45% compared with CK (*p* < 0.05), respectively. Obviously, MeJA treatment and ABA treatment had a more positive impact on the content of bioactive components in *A. argyi* leaves and, in particular, ABA treatment also greatly increased the content of jaceosidin (flavonoid).

### 3.3. The Correlation between the Elements and the Bioactive Components

**Figure 5:**
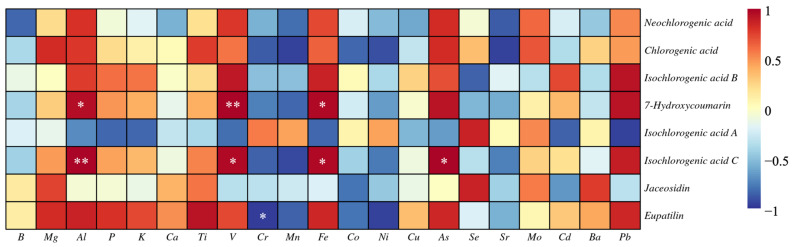
Correlation heat map between elements and bioactive compounds of *A. argyi*. * indicates a significant correlation (*p* < 0.05). ** indicates a significant correlation (*p* < 0.01).

We further analyzed the correlation between the content of elements and the content of bioactive components, and the results are shown in Figure 5. The correlation between different elements and bioactive components shows obvious differences. 7-hydroxycoumarin was significantly positively correlated with Al (*p* < 0.05), V (*p* < 0.01) and Fe (*p* < 0.05). Isochlorogenic acid C was significantly positively correlated with Al (*p* < 0.01), V (*p* < 0.05), Fe (*p* < 0.05) and As (*p* < 0.05). Eupatilin was significantly negatively correlated with Cr (*p* < 0.05). Correlation analysis showed that the difference in the absorption of elements by *A. argyi* leaves affected the synthesis of bioactive components.

### 3.4. Widely Untargeted Metabolomic Analysis of A. argyi Leaves

**Figure 6:**
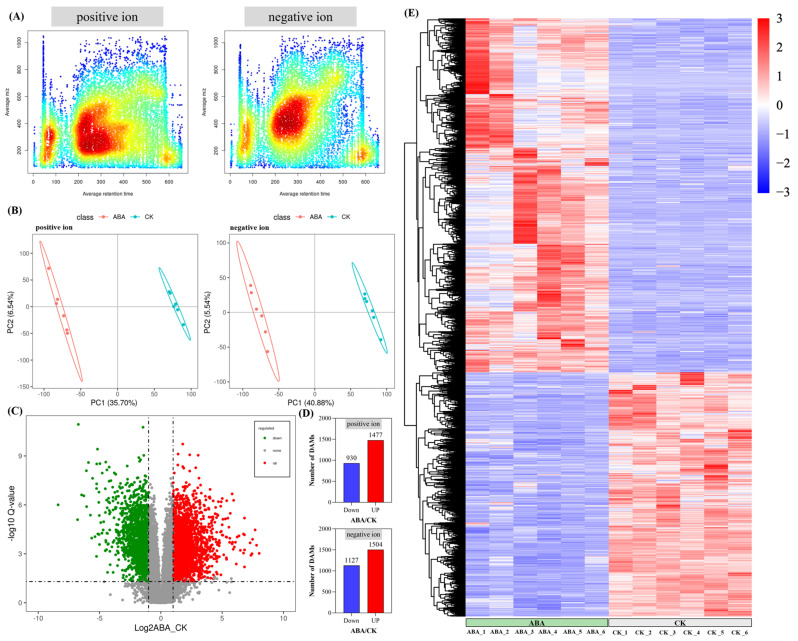
Basic metabolite information of ABA and CK based on widely untargeted metabolomic analysis. (**A**) m/z-RT distribution of metabolites. (**B**) PLS-DA score plot of metabolites. (**C**) Significance analysis of the DAMs between the ABA and CK by volcano plot. (**D**) Number of DAMs between ABA and CK. (**E**) ABA regulated the metabolites of *A. argyi* leaves.

According to our previous analysis, ABA had a more positive effect on phenylpropanoids and flavonoids in *A. argyi* leaves. Therefore, the widely untargeted metabolomic analysis by UPLC-MS/MS aimed at deciphering the effects of ABA on the metabolites in *A. argyi* leaves. Basic metabolite information of ABA (*n* = 6) and CK (*n* = 6) based on widely untargeted metabolomic analysis is shown in Figure 6. The m/z of the positive ions was concentrated between 150 and 600, and the retention time was concentrated between 200 and 400. The m/z of the negative ions was concentrated between 200 and 600, and the retention time was concentrated between 200 and 350 (Figure 6A). The partial least squares discriminant analysis (PLS-DA) was used to present the overall difference in metabolites between ABA and CK (Figure 6B). The clear distinction of metabolite expression between ABA and CK lays the foundation for the DAM identification. Significance analysis of the DAMs between ABA and CK is shown in Figure 6C,D. A total of 5038 identified DAMs with 2407 positive ions (1477 significantly up-regulated DAMs and 930 significantly down-regulated DAMs) and 2631 negative ions (1504 significantly up-regulated DAMs and 1127 significantly down-regulated DAMs) were found. The expression heatmap of DAMs is shown in Figure 6E, in which ABA shows a positive stimulating effect on the production of metabolites with a total of 2981 significantly up-regulated DAMs and a total of 2057 down-regulated DAMs. Therefore, the next analysis focuses on these metabolites stimulated by ABA.

**Figure 7:**
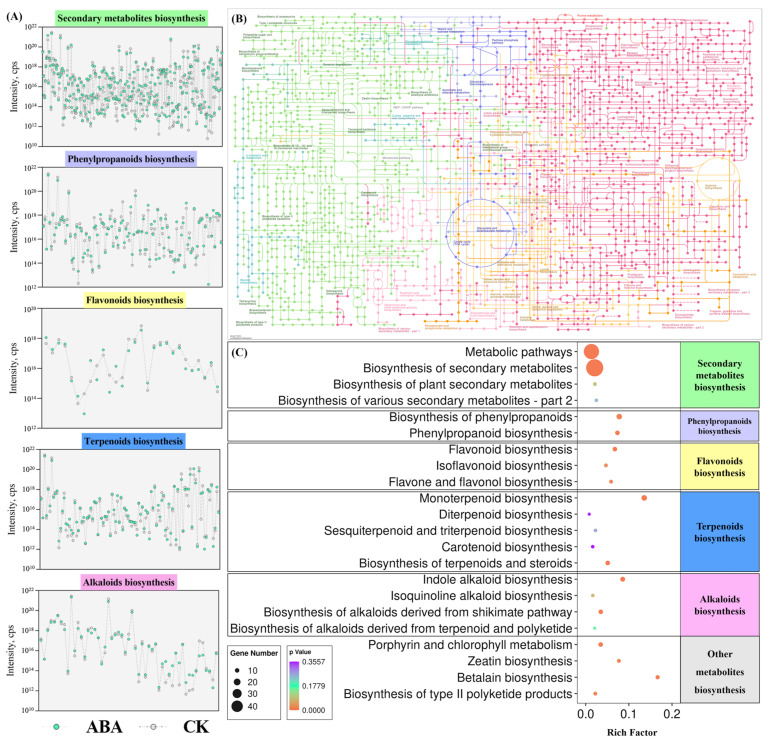
Identification and KEGG annotation of the DAMs between ABA and CK. (**A**) The abundances of the DAMs belonging to different metabolic categories. (**B**) Overview of the metabolic pathways of DAMs between ABA and CK. (**C**) Statistical analysis of DAMs enriched in the secondary metabolite biosynthesis pathways by KEGG annotation.

After identification and KEGG annotation of DAMs, we mainly selected the various metabolites involved in secondary metabolite activities of *A. argyi* leaves for analysis. The DAMs are assigned to different secondary metabolite categories, mainly including phenylpropanoids, flavonoids, terpenoids and alkaloids (Figure 7). The normalized ion intensity of each DAM belonging to different secondary metabolite activities was calculated (Figure 7A). An overview of the secondary metabolic pathways of DAMs between ABA and CK is presented in Figure 7B,C and shows the statistical analysis of DAMs enriched in secondary metabolite biosynthesis pathways using KEGG annotation. After ABA treatment, *A. argyi* leaves showed a tendency of active biosynthesis of secondary metabolites. In the next step, the DAMs annotated to different secondary metabolites biosynthesis pathways need to be analyzed further.

**Figure 8:**
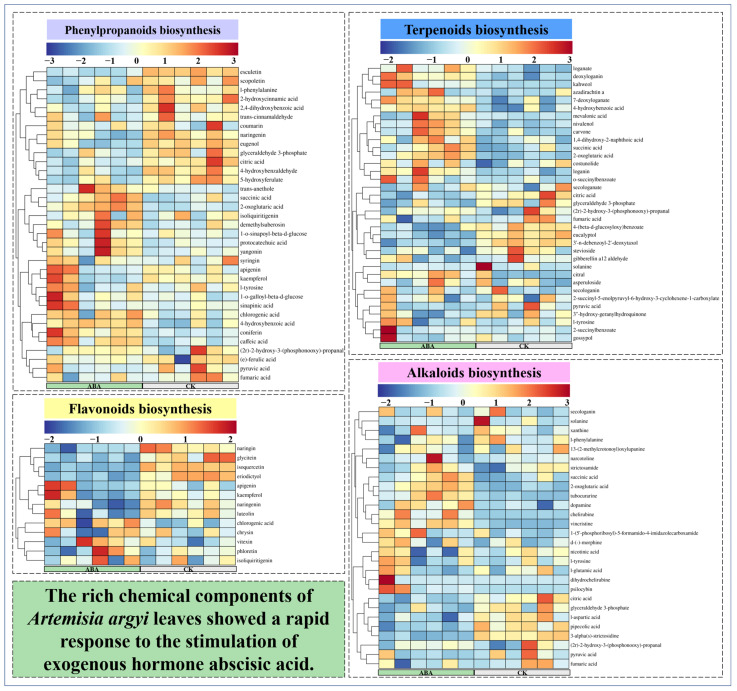
A heatmap of the relative amounts of phenylpropanoid, flavonoid, terpenoid and alkaloid biosynthesis.

Medicinal plants have attracted much attention because of their abundant secondary metabolites. In this study, 35 metabolites involved in phenylpropanoid biosynthesis, 13 metabolites involved in flavonoid biosynthesis, 35 metabolites involved in terpenoid biosynthesis and 28 metabolites involved in alkaloid biosynthesis were identified (Figure 8). The rich chemical components of *A. argyi* leaves showed a rapid response to the stimulation of the exogenous hormone ABA. The metabolites that were most up-regulated in the phenylpropanoid biosynthesis pathway were coumarin, 1-O-sinapoyl-*β*-D-glucose, l-phenylalanine, succinic acid and sinapinic acid. The metabolite that was most up-regulated in the flavonoid biosynthesis pathway was chlorogenic acid. More flavonoid metabolites were significantly down-regulated after ABA treatment. The metabolites that were most up-regulated in the terpenoid biosynthesis pathway were citral, loganin, secologanin and deoxyloganin. The metabolites that were most up-regulated in the alkaloid biosynthesis pathway were psilocybin, dihydrochelirubine, chelirubine, vincristine, tubocurarine and secologanin. The above analysis shows that ABA changes the metabolic direction of secondary metabolites in *A. argyi* leaves and stimulates the biosynthesis of multiple secondary metabolites, including phenylpropanoids, flavonoids, terpenoids, alkaloids and others.

**Figure 9:**
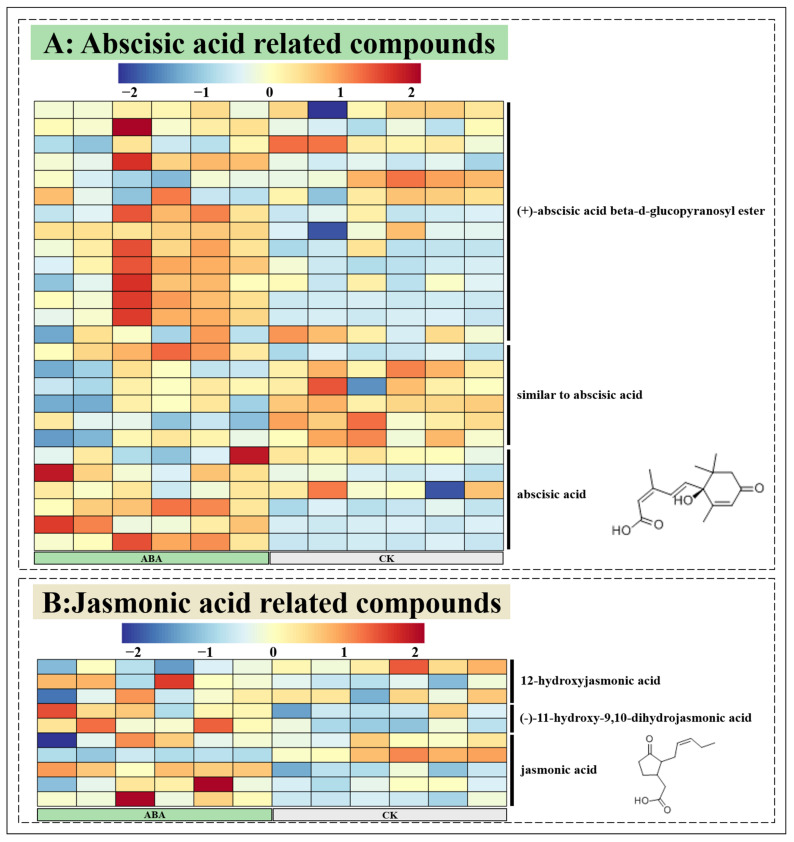
A heatmap of the relative compounds of abscisic acid and jasmonic acid.

In order to determine whether exogenous ABA has an effect on the synthesis of phytohormones in *A. argyi* leaves, we annotated the related compounds of ABA, JA and SA. In this study, only ABA and JA-related compounds were annotated, and no SA-related compounds were annotated (Figure 9). Significant amounts of (+)-abscisic acid β-D-glucopyranosyl ester were found among the annotated ABA-related compounds (Figure 9A). (+)-abscisic acid β-D-glucopyranosyl ester accumulated in plants can make free ABA form rapidly under abiotic stress. In addition, a large amount of ABA was also observed in the tissue of *A. argyi* leaves after ABA treatment. JA-related compounds are shown in Figure 9B, including 12-hydroxyjasmonic acid, (−)-11-hydroxy-9,10-dihydrojasmonic acid and jasmonic acid. However, these compounds did not show regular changes after ABA treatment. The above results showed that exogenous ABA had no significant effect on JA in *A. argyi* leaves, but could promote the synthesis of ABA.

## 4. Discussion

At present, there is still a lack of understanding about the element absorption and the changes in secondary metabolic components in *A. argyi* under the treatments of exogenous hormones. In this work, we analyzed the difference in 21 elements absorbed by *A. argyi* leaves under three exogenous hormone (MeJA, SA and ABA) treatments, and also clarified the correlation between elements and bioactive components under different exogenous hormone treatments. The mechanism of the effect of ABA on the formation of secondary metabolic components in *A. argyi* leaves was further investigated by widely untargeted metabolomic analysis. These results provide a new perspective for the changes in element absorption and the mechanism of secondary metabolic components in *A. argyi* leaves under exogenous hormone treatments, and also deepen people’s understanding of the interaction mechanism between medicinal plants and hormones.

Plant hormones are organic signal molecules that can produce obvious physiological effects at very low concentrations, and play a crucial role in plant growth and development [27]. In the process of plant growth and development, elements, as a kind of basic component that cannot be synthesized by plants themselves, are usually taken by plants from the soil, water and atmosphere in the growth environment and enter the plants to participate in various life activities [28,29]. Therefore, it is of great significance to determine the change in element content in *A. argyi* leaves under exogenous hormone treatments. At present, the determination methods of inorganic elements mainly include atomic absorption spectrometry (AAS), atomic fluorescence spectrometry (AFS), inductively coupled plasma atomic emission spectrometry (ICP-AES), inductively coupled atomic emission spectrometry (ICP-OES), inductively coupled plasma mass spectrometry (ICP-MS), etc. [30,31]. ICP-MS is widely used because of its high sensitivity, low detection limit, less interference and the ability to simultaneously determine multiple elements of different content levels [32]. In this study, we used ICP-MS technology to determine the changes in 21 elements (including four non-metallic elements and 17 metal elements) in *A. argyi* leaves under different exogenous hormone treatments. The results showed that the absorption and enrichment of elements by *A. argyi* leaves were significantly different under exogenous hormone treatments. The content of 21 elements in *A. argyi* leaves from high to low was K > Mn > B > Ca > Sr > Mg > P > Cu > Cr > Ni > Al > Fe > Mo > Ti > V >Ba > Pb > Cd > Se > As > Co. The content of K in *A. argyi* leaves is the highest among the 21 determined elements. K is the activator of many enzymes (more than 60 kinds) in the organism and an important component of cell osmotic potential [33]. It has the functions of regulating the opening and closing of stomata, promoting photosynthetic phosphorylation and promoting the transport of assimilates [34]. The content of K decreased significantly under the treatment of three hormones (*p* < 0.05), which may affect the physiological function of *A. argyi* leaves. Mn is a component of chloroplasts, which promotes seed development and early seedling growth, and plays an important role in photosynthesis and protein formation [35]. After treatment with three hormones, the absorption of Mn by *A. argyi* leaves changed significantly. B affects the development of plant reproductive organs and the elongation and division of cells, and plays an important role in flowering and fruiting [36]. In addition to the elements with high content, some elements with very low content also changed under different exogenous hormone treatments. Our results show that different exogenous hormone treatments changed the absorption and enrichment of elements in *A. argyi* leaves, and their composition also changed significantly.

The growth and development of plants cannot be separated from the soil [37]. The composition and content of various elements in the soil directly affect the growth and development and the types and quantities of secondary metabolites in medicinal plants [38]. As found in a study on black and green teas (*Camellia sinensis*) and erva mate (*Ilex paraguariensis*), Zn is highly correlated with flavonoids in leaves [39]. A study on 92 *Scutellaria baicalensis* and soil samples found that excessive element content in soil was not conducive to the accumulation of chemical components in *S. baicalensis* [40]. In a study on the correlation between *Bupleurum chinense* (including wild type and cultivated type) with 29 habitat and soil factors, it was found that there was a significant positive correlation between Fe and *saikosaponins* (*p* < 0.01), and a significant negative correlation between Cu, Mg and *saikosaponins* (*p* < 0.05) [41]. In this study, we first identified the effects of three exogenous hormones on eight bioactive components (six phenylpropanoids and two flavonoids). MeJA treatment significantly increased the content of neochlorogenic acid, chlorogenic acid, 7-hydroxycoumarin and isochlorogenic acid C in *A. argyi* leaves. After SA treatment, only the content of isochlorogenic acid A increased significantly. ABA treatment not only increased the content of phenylpropanoid compounds (neochlorogenic acid, chlorogenic acid and isochlorogenic acid A), but also increased the content of flavonoid (jaceosidin). We further used correlation analysis to link elements and secondary metabolites in *A. argyi* leaves under different hormone treatments. 7-hydroxycoumarin was significantly positively correlated with Al (*p* < 0.05), V (*p* < 0.01) and Fe (*p* < 0.05). Isochlorogenic acid C was significantly positively correlated with Al (*p* < 0.01), V (*p* < 0.05), Fe (*p* < 0.05) and As (*p* < 0.05). Eupatilin was significantly negatively correlated with Cr (*p* < 0.05). Elements are the material basis for plant growth and development and the formation of nutritional quality. These elements can participate in different plant life activities (such as chlorophyll formation and plant growth) [42]. There may be three reasons for the relationship between elements and bioactive components in *A. argyi* leaves: (1) under exogenous hormone treatments, the absorption of elements in *A. argyi* leaves changed, resulting in changes in plant secondary metabolism; (2) elements participate in the composition of enzymes in plant tissues, and biologically active components undergo decomposition or synthesis reactions due to changes in enzyme activity; (3) exogenous hormones and absorbed elements may participate in regulating the expression of genes related to biosynthetic pathways in *A. argyi* leaves, thus affecting the content of bioactive components. However, confirmation of these possible reasons will require us to conduct further experiments in future research. In our work, these results indicated that when *A. argyi* was stimulated by exogenous hormones, the content of secondary metabolites is adjusted in the leaves through changes in the absorption and enrichment of elements, which was a response to exogenous hormone regulation.

Metabolites are the end products of cellular regulatory processes, and their levels can be viewed as the ultimate response of biological systems to genetic or environmental changes [43]. The metabolites synthesized by a biological system constitute its “metabolome”, which mainly refers to the collection of all low molecular weight metabolites in the cell [44]. These molecules are participants in general metabolic reactions and are necessary for the maintenance of growth and normal function of the cell. As an important branch of systems biology, metabonomics is an interdisciplinary subject that has developed rapidly following genomics, transcriptomics and proteomics [45]. In recent years, it has attracted extensive attention in the field of plant research, especially in the field of medicinal plants that targets secondary metabolites [46]. Metabonomics has become an effective method to deeply analyze the relationship between changes in plant histochemical components and exogenous hormones [47]. In this study, the widely untargeted metabolome was used to analyze the changes in metabolites of *A. argyi* leaves under ABA treatment. ABA treatment had a significant effect on the distribution and species of metabolites. After filtering, 5038 high-quality metabolites were used to screen the DAMs (2407 positive ions and 2631 negative ions). After identification and KEGG annotation of DAMs, the DAMs were assigned to different secondary metabolites categories, mainly including phenylpropanoids, flavonoids, terpenoids and alkaloids. After ABA treatment, *A. argyi* leaves showed a tendency of active biosynthesis of secondary metabolites. A total of 35 metabolites involved in phenylpropanoid biosynthesis, 13 metabolites involved in flavonoid biosynthesis, 35 metabolites involved in terpenoid biosynthesis and 28 metabolites involved in alkaloid biosynthesis were identified. In order to determine whether exogenous ABA has an effect on the synthesis of phytohormones in A. argyi leaves, we annotated the related compounds of ABA, JA and SA. We found that exogenous ABA had no significant effect on JA in *A. argyi* leaves, but could promote the synthesis of ABA. At this time, ABA changes from the bound state to the free state to exert its physiological activity in *A. argyi* leaves. The above analysis shows that ABA changes the metabolic direction of secondary metabolites in *A. argyi* leaves and stimulates the biosynthesis of multiple secondary metabolites including phenylpropanoids, flavonoids, terpenoids, alkaloids and others.

## 5. Conclusions

In this study, we discussed the regulation of exogenous hormones on the absorption of elements and the accumulation of secondary metabolites in leaves of the medicinal plant *A. argyi*. The content of 21 elements in *A. argyi* leaves from high to low was K > Mn > B > Ca > Sr > Mg > P > Cu > Cr > Ni > Al > Fe > Mo > Ti > V > Ba > Pb > Cd > Se > As > Co. Different exogenous hormone treatments changed the absorption and enrichment of elements in *A. argyi* leaves, and the composition also changed significantly. MeJA treatment significantly increased the content of neochlorogenic acid, chlorogenic acid, 7-hydroxycoumarin and isochlorogenic acid C in *A. argyi* leaves (*p* < 0.05). After SA treatment, only the content of isochlorogenic acid A increased significantly (*p* < 0.05). ABA treatment not only increased the content of neochlorogenic acid, chlorogenic acid and isochlorogenic acid A, but also increased the content of jaceosidin (*p* < 0.05). When *A. argyi* was stimulated by exogenous hormones, the content of secondary metabolites was adjusted in the leaves through changes in the absorption and enrichment of elements. The widely untargeted metabolomic analysis further confirmed that ABA changes the metabolic direction of secondary metabolites in *A. argyi* leaves and stimulates the biosynthesis of multiple secondary metabolites, including phenylpropanoids, flavonoids, terpenoids, alkaloids and others. These results provide a new perspective for the changes in element absorption and the mechanism of secondary metabolic components in *A. argyi* leaves under exogenous hormone treatments, and also deepen people’s understanding of the interaction mechanism between medicinal plants and hormones.

## Data Availability

The data presented in this study are available on request from the corresponding author. The data are not publicly available due to the corresponding author needs further research.

## Supplementary Materials

The following supporting information can be downloaded at: https://www.mdpi.com/article/10.3390/metabo12100984/s1, Table S1: The linear relationship of 21 elements; Table S2: The linear relationship of 8 bioactive components.
