# Supplementary material for: Study on the Regulation of Exogenous Hormones on the Absorption of Elements and the Accumulation of Secondary Metabolites in the Medicinal Plant Artemisia argyi Leaves
Source: Metabolites. 2022 Oct 17;12(10):984. doi: 10.3390/metabo12100984 (PMC9609755; doi:10.3390/metabo12100984)
Supplement: Supplementary file 1 [file metabolites-12-00984-s001.zip › Supplementary Material. Table S1.pdf]

Table S1 The linear relationship of 21 elements.

Element	Regression equation	R^2
B	Y=17780.591X+342674.821	0.996
Na	Y=82720.896X+8018782.106	0.987
Mg	Y=92384.176X+1581598.407	0.991
Al	Y=66405.145X+6890877.427	0.999
P	Y=5205.398X+809029.403	0.991
K	Y=84914.212X+6133610.229	0.990
Ca	Y=5497.665X+827975.725	0.969
Ti	Y=124488.712X+209415.748	0.999
V	Y=133712.848X+36221.520	0.996
Cr	Y=125034.490X+125467.126	1.000
Mn	Y=218205.035X+209991.832	1.000
Fe	Y=4403.239X+226236.594	0.986
Co	Y=150401.167X+11099.937	1.000
Ni	Y=30029.727X+26897.907	1.000
Cu	Y=71366.058X+104663.907	1.000
As	Y=16056.110X+4768.524	0.999
Se	Y=1208.536X+3483.118	1.000
Sr	Y=284365.300X+109939.198	1.000
Mo	Y=38054.183X+14542.157	1.000
Cd	Y=23041.150X+708.374	1.000
Sn	Y=83646.168X+6709.425	1.000
Ba	Y=46525.293X+102227.507	0.997
Pb	Y=203730.475X+1284501.449	1.000
