# Supplementary material for: Study on the Regulation of Exogenous Hormones on the Absorption of Elements and the Accumulation of Secondary Metabolites in the Medicinal Plant Artemisia argyi Leaves
Source: Metabolites. 2022 Oct 17;12(10):984. doi: 10.3390/metabo12100984 (PMC9609755; doi:10.3390/metabo12100984)
Supplement: Supplementary file 1 [file metabolites-12-00984-s001.zip › Supplementary Material. Table S2.pdf]

Table S2 The linear relationship of 8 bioactive components

	Regression equation	R^2	Linear range (μg·L^{-1})
Neochlorogenic acid	Y=25.673x-13.855	0.9994	0.625~20.0
Chlorogenic acid	Y=28.850x-14.752	0.9996	0.631~20.2
Isochlorogenic acid B	Y=18.276x-18.041	0.9993	5.000~30.0
7-hydroxycoumarin	Y=48.606x-22.503	0.9995	0.619~19.8
Isochlorogenic acid A	Y=23.364x-14.643	0.9996	0.631~20.2
Isochlorogenic acid C	Y=40.508x-27.437	0.9993	0.619~19.8
Jaceosidin	Y=38.460x-188.882	0.9996	0.631~20.2
Eupatilin	Y=53.694x-25.287	0.9995	0.612~19.6
